# Rapid slice-to-volume four-dimensional flow in pediatric congenital heart disease: a feasibility study

**DOI:** 10.1016/j.jocmr.2025.101887

**Published:** 2025-03-24

**Authors:** Valérie Béland, Datta Singh Goolaub, Sharon Portnoy, Shi-Joon Yoo, Christopher Z. Lam, Christopher K. Macgowan

**Affiliations:** a Translational Medicine, The Hospital for Sick Children, Toronto, Ontario, Canada; b Medical Biophysics, University of Toronto, Toronto, Ontario, Canada; c Department of Diagnostic Imaging, The Hospital for Sick Children, Toronto, Ontario, Canada

**Keywords:** Slice-to-volume reconstruction, Cardiovascular imaging, 4D flow, Pediatrics

## Abstract

**Background:**

Cardiovascular magnetic resonance (CMR) allows cardiac hemodynamic assessment in patients with congenital heart disease (CHD). However, conventional techniques are time-consuming and may require blood contrast agents. Slice-to-volume reconstruction (SVR) four-dimensional (4D) flow is an innovative imaging technique that may overcome these limitations. This study aimed to assess the feasibility of SVR 4D flow in pediatric CHD.

**Methods:**

Patients with CHD (n = 7, age = 12.9 ± 2.8 years) underwent CMR with conventional two-dimensional (2D) phase-contrast magnetic resonance imaging (2D PCMRI) and SVR 4D flow. SVR 4D flow datasets were reconstructed from multi-slice 2D spiral PCMRI acquisitions, which were combined via slice-to-volume reconstruction. Mean flows in major thoracic vessels were measured and compared between the two techniques. Signal-to-noise ratio (SNR) and contrast-to-noise ratio (CNR) were calculated for each participant and compared between imaging techniques.

**Results:**

Linear regression for SVR 4D flow against 2D PCMRI showed good agreement for mean flows (slope = 1.03, intercept = −5.31 mL/s, *r*^2^ = 0.95). The SNR and CNR did not differ significantly between 2D PCMRI and SVR 4D flow data (SNR: *p = *0.85, CNR: *p = *0.90).

**Conclusion:**

Our results suggest that SVR 4D flow CMR is a feasible 5-minute scan (relative to multiple 2D PCMRI prescriptions and scans) in pediatric patients with CHD. SVR 4D flow showed good agreement with 2D PCMRI for mean flow measurements. The advantages of SVR 4D flow support further research such as its comparison with conventional 4D flow.

## Graphical abstract

**Figure:**
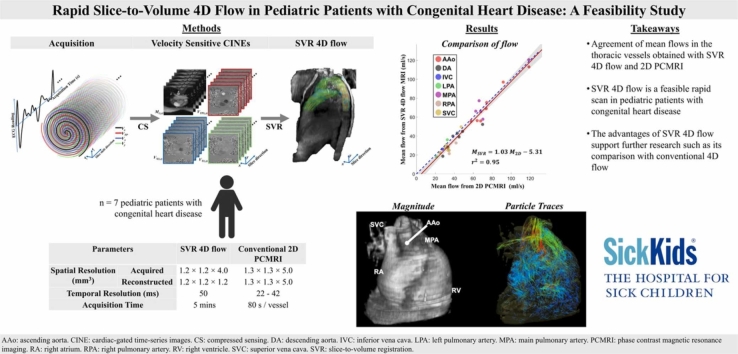

## 1. Introduction

Phase-contrast magnetic resonance imaging (PCMRI) is essential for comprehensive assessment of critical congenital heart disease (CHD). Critical CHD includes life-threatening malformations of the heart and major vessels and must be treated soon after birth. Traditionally, PCMRI is performed using a two-dimensional (2D) slice across the target vessel. Modern advances now enable four-dimensional (4D) flow in these patients, eliminating the need for multiple plane prescriptions to assess complex morphologies [1]. A potential limitation of 4D flow is its lack of inflow enhancement, which may reduce blood vessel conspicuity, challenging anatomical segmentation and clinical analysis. To improve the signal-to-noise ratio (SNR) for 4D flow MRI, intravenous contrast agents are commonly used, but are associated with additional costs and risks [2].

An alternative 4D flow approach has recently been demonstrated that overcomes these limitations by using a 2D multiplanar acquisition [3]. In this approach, 2D PCMRI acquisitions obtained in a short 5-minute scan are combined with slice-to-volume reconstruction (SVR) to generate 4D data. Since the technique is based on 2D PCMRI, SNR (in blood) naturally benefits from inflow enhancement without the addition of contrast agents. Previously, this method was demonstrated with multi-slice radial PCMRI in fetal subjects. Here, we test the feasibility of this approach postnatally in a small cohort of pediatric patients with CHD, but using a continuous radiofrequency shift to create a moving slice (referred to as “SWEEP”) and a spiral acquisition [4]. This approach is hereafter referred to as SVR 4D flow.

## 2. Theory

### 2.1 Acquisition and reconstruction

**Fig. 1:**
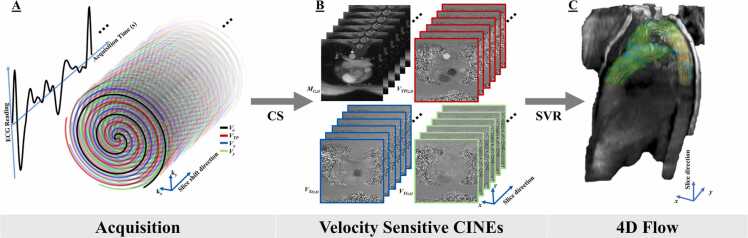
Acquisition and reconstruction of spiral multidimensional PCMRI data to generate a flow-sensitive volume. (A) Spiral PCMRI is acquired with a continuous small shift in the slice direction. (B) Consecutive spiral data are grouped to generate velocity-sensitive CINEs with CS. (C) CINEs are combined into dynamic, sensitive flow volumes using slice-to-volume reconstruction. Velocity acquisitions are color-coded: flow compensation: *V*_*o*_ (black), Through-plane flow: *V*_*TP*_ (red), In-plane flow along *x*-dimension: *V*_*X*_ (blue), and In-plane flow along *y*-dimension: *V*_*Y*_ (green). *CS* compressed sensing, *CINEs* cardiac-gated time-series images, *M* magnitude, *(z,t) s*lice position *z* and time series *t, SVR s*lice-to-volume reconstruction, *PCMRI* phase-contrast magnetic resonance imaging

The proposed SVR 4D flow approach involves the acquisition of accelerated 2D spiral PCMRI with multidimensional flow encoding. The acquisition interleaves velocity encoding directions while continuously updating the trajectory angle by the golden angle and includes a small continuous spatial shift in the slice direction at each *TR* (Fig. 1A) [4], [5]. The continuous shift helps maintain a steady state over the region being imaged, enabling retrospective reconstruction of 2D slices from windows of consecutively acquired measurements. With step size being much smaller than the slice thickness, there is dense sampling in the slice direction, facilitating retrospective volumetric reconstructions at high isotropic resolution using SVR.

Electrocardiogram (ECG)-gated 2D flow-sensitive CINEs are generated from the multidimensional PCMRI data using compressed sensing (CS) with temporal total variation (TTV) (Fig. 1B) [7]. The multiplanar CINEs are then combined into an isotropic dynamic volume using SVR to yield volumetric flow [3,8] (Fig. 1C).

## 3. Materials and methods

### 3.1 Participants

This cross-sectional study was approved by the Research Ethics Board (REB #1000071424). Inclusion criteria included children 0–18 years old with a diagnosis of CHD scheduled for a clinical cardiac MRI requiring 2D PCMRI flow assessment. Consenting participants had the SVR 4D flow sequence added to their clinical scan. Exclusion criteria included refusal of consent, refusal of assent by legal guardian/patient, and/or contraindications to MRI.

### 3.2 Data acquisition

Imaging was performed on a 1.5T MRI Avanto^FIT^ (Siemens Healthineers, Erlangen, Germany) under free breathing conditions, with the ECG signal recorded for retrospective cardiac gating. Each participant underwent both conventional 2D PCMRI and SVR 4D flow during a 1-hour examination. Conventional 2D PCMRI was performed as clinically indicated for assessment of target vessels as follows: the ascending aorta (AAo), descending aorta (DA), main pulmonary artery (MPA), right/left pulmonary artery (RPA/LPA), and/or superior/inferior vena cava (SVC/IVC). Planning of 2D PCMRI scan planes took approximately 30 s per vessel and was prescribed by MRI technologists. The SVR 4D flow acquisition (axial plane) was prescribed over a volume spanning the heart and aortic arch with a continuous spatial shift of 0.005 mm/TR along the slice direction. Parameters for these sequences were as follows. For conventional 2D PCMRI, acquired under free breathing: spatial resolution = 1.3 × 1.3 × 5.0 mm^3^, echo time and repetition time (TE/TR) ∼3.0/5.3 ms, number of segments = 2–4 (heart-rate dependent to achieve a temporal resolution of approximately 25 cardiac phases per cycle), temporal resolution = 22–42 ms (heart-rate dependent), acceleration = GRAPPA × 2, number of averages = 2, acquisition time∼80 s/vessel, velocity encoding sensitivity (VENC) = 100–400 cm/s, and flip angle = 20°. For SVR 4D flow, also under free breathing: acquired spatial resolution = 1.2 × 1.2 × 4.0 mm^3^ (for isotropic SVR reconstruction at 1.2 × 1.2 × 1.2 mm^3^ resolution), TE/TR = 3.0/7.5 ms, temporal resolution∼50 ms, acceleration = 12, acquisition time = 5 min, VENC = 150 cm/s, and flip angle = 15°. The flip angle for SVR 4D flow was less than 2D PCMRI because it was previously optimized for contrast-to-noise ratio (CNR) and CS outcomes.

If clinically indicated, contrast agent (Gadobutrol, Gadovist®, Bayer Healthcare, Berlin, Germany) was administered at the beginning of the examination, but not for flow assessment. To evaluate the potential influence of contrast agent on SVR 4D flow and 2D PCMRI scans, SNR and CNR were calculated in each participant. SNR was calculated as the mean signal of the vessel lumen: AAo and (SVC or DA) divided by the (corrected) standard deviation of background noise in air. CNR was calculated as the absolute difference between the vessel signal and nearby lung parenchyma, divided by background noise.

### 3.3 Reconstruction

Using the spiral multidimensional SWEEP PCMRI data, retrospectively gated CINE reconstructions were performed as described in the Theory section using precompiled MATLAB functions (MathWorks, Natick, Massachusetts). These were computed using slices comprising 996 spiral arms with 332 arms shared between adjacent slices. CS reconstruction was performed for 30 iterations with a TTV weighting of 0.08 and trajectory correction based on the gradient impulse response function [9]. SVR was performed with an edge-preserving spatial regularization weight of 0.01 for 10 iterations [8].

### 3.4 Flow analysis

2D PCMRI data were analyzed in cvi42 (Version 5.14.2, Circle Cardiovascular Imaging, Canada). SVR 4D flow data were analyzed in the prototype software 4D Flow v2.4 (Siemens Healthineers, Germany), because it provided convenient visualization options that facilitated segmentation [10]. In each analysis software, target vessel borders were contoured manually, automatically propagated to all phases, and edited if necessary. Background phase correction and automatic anti-aliasing were applied.

### 3.5 Statistical analysis

Statistical analysis was performed using RStudio (Version 2024.12.1, R Foundation for Statistical Computing, Vienna, Austria). A linear mixed model using restricted maximum likelihood (REML) examined the effects of imaging technique, vessel type, and contrast agent (fixed effects) on SNR or CNR values, with participant included as a random effect to account for individual differences. Mean flows in the great vessels were compared by linear regression and Bland–Altman analysis. A linear mixed model using REML examined the effects of imaging technique and vessel type (fixed effects) on mean flows, with participant included as a random effect to account for individual differences. The significance of the fixed effects was assessed with a Type III analysis of variance for each linear mixed model.

## 4. Results

### 4.1 Participants and MRI techniques

Informed consent was obtained from seven children/adolescents meeting inclusion criteria during the recruitment window (Table 1). 2D PCMRI was acquired for clinical assessment from the AAo, DA, RPA, LPA, and SVC of all participants (P), whereas the MPA was acquired in six and the IVC in one. SVR 4D flow data were obtained in all seven participants; however, inadequate slice prescription resulted in incomplete coverage of the LPA and/or RPA in four cases (P3, P4, P5, and P7), and MPA and SVC in one case (P7). Further, velocity aliasing in the RPA of P1 could not be corrected, preventing flow quantification. For both 2D and 4D acquisitions, the LPA of P1 and MPA of P5 could not be reliably quantified because of artifact from nearby metallic implants. The remaining data provided 31 pairs (AAo = 7, DA = 7, MPA = 5, LPA = 2, RPA = 3, SVC = 6, IVC = 1) of mean flow by both 2D PCMRI and SVR 4D flow for comparison.

**Table 1:** Summary of diagnosis, age, and sex of participants.

Participants	Primary diagnosis	Age (years)	Sex
P1	Repaired tetralogy of Fallot	17	Male
P2	Unrepaired Ebstein anomaly	12	Female
P3	Critical PS sp transannular patch	13	Female
P4	Tetralogy of Fallot sp pulmonary valvotomy, and MPA patch	12	Male
P5	Tetralogy of Fallot / PA sp Melody PVR, bilateral SVC, RUPV PAPVC	15	Male
P6	Repaired tetralogy of Fallot	13	Male
P7	Williams syndrome sp aortic reconstruction	8	Female
	-	12.9±2.8	-

*MPA* main pulmonary artery, *PAPVC* partial anomalous pulmonary venous connection, *PA* pulmonary atresia, *PS* pulmonary stenosis, *PVR* pulmonary valve replacement, *RUPV* Right upper pulmonary vein, *sp* status post-procedure/surgery, *SVC* superior vena cava.

Data are presented as means +/- standard deviation.

The SNR and CNR were not significantly different between 2D PCMRI and SVR 4D flow [SNR: *F*(1, 17.81) = 0.04, *p = *0.85, CNR: *F*(1, 17.82) = 0.017, *p = *0.90], with mean (standard deviation [SD]) values for 2D PCMRI [SNR: 35.80 (6.70), CNR: 30.73 (6.39)] and for SVR 4D flow [SNR: 36.18 (10.84), CNR: 30.97 (10.43)]. The contrast agent injected in three participants (P3, P6, and P7) at the beginning of their scan did not significantly affect the SNR (*p *= 0.90) or CNR (*p *= 0.89), and the vessel type did not significantly affect the SNR (*p *= 0.14) or CNR (*p *= 0.17). In both SNR and CNR analyses, the participant (random effect) contributed dramatically to the observed total variances (79% each), possibly attributable to coil coverage and participant size. Variances (SDs) themselves were [SNR: 99.82 (9.99), CNR: 91.11 (9.55)]. The reconstruction pipeline for SVR 4D flow took approximately 4 h per patient. Segmentation and flow analysis took approximately 30 min each for SVR 4D flow and 2D PCMRI.

### 4.2 Flow accuracy

**Fig. 2:**
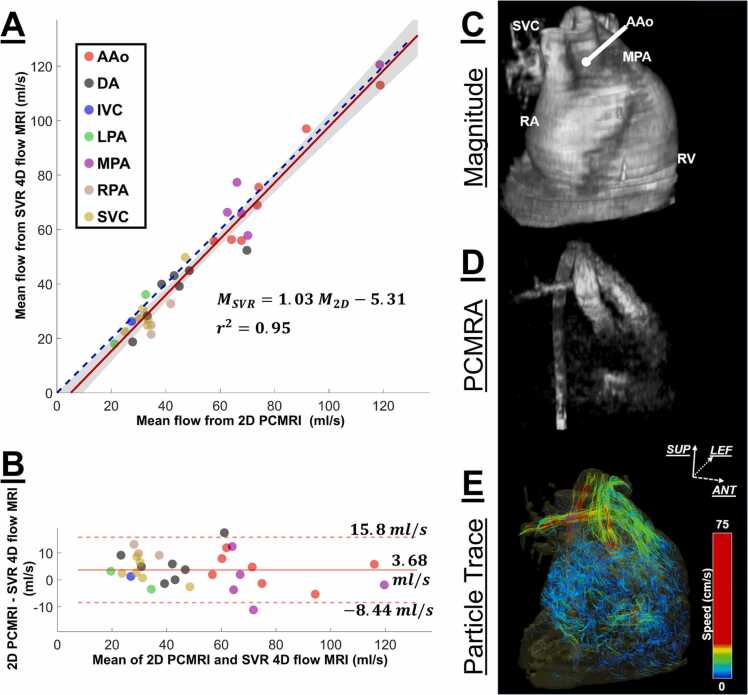
Flow comparisons between SVR 4D flow and 2D PCMRI, along with volumetric visualization of SVR 4D flow. (A) Linear regression for the comparison between mean flow measurements from SVR 4D flow and 2D PCMRI in the great vessels of seven pediatric patients with congenital heart disease. The solid red line represents the fitted line to the data, the gray line represents the 95% confidence interval, and the blue dashed line represents the identity line. (B) Bland–Altman represents the difference in the mean flow between 2D PCMRI and SVR 4D flow against the mean of the mean flow measurements of both techniques. The solid red line represents the bias, and the dashed red lines represent the limits of agreement. (C-E) Volumetric visualizations of a patient’s heart with Ebstein’s anomaly at systole in a lateral view. An enlarged right ventricle is observed in the 3D rendering of the magnitude image (C). Blood ejection through the AAo and MPA can be seen in the maximum intensity projection image of the PCMRA (D) and particle trace image (E). *AAo* (red) ascending aorta, *DA* (gray) descending aorta, *IVC* (purple) inferior vena cava, *LPA* (green) left pulmonary artery, *MPA* (magenta) main pulmonary artery, *PCMRA* phase-contrast magnitude resonance angiography, *RPA* (brown) right pulmonary artery, *SVC* (gold) superior vena cava, *M_SVR_* mean flow measurement from SVR 4D flow, *M_2D_* mean flow measurement from 2D PCMRI, *PCMRI* phase-contrast magnetic resonance imaging

A linear regression (Fig. 2A) computed for 31 pairs of mean flow measurements showed good agreement between SVR 4D flow and 2D PCMRI with slope = 1.03 (95% confidence interval = [0.94 1.12]), intercept = −5.31 mL/s (95% confidence interval = [−10.70 0.04] mL/s), and *r*^2^ = 0.95. The Bland–Altman analysis (Fig. 2B) had a bias of 3.68 mL/s and limits of agreement [−8.44 15.80] mL/s. The analysis of variance showed that the imaging techniques (SVR 4D flow and 2D PCMRI) do not have a significant effect on the mean flow values, *F*(1, 47.97) = 3.11, *p = *0.08, whereas the vessel type significantly affects the mean flow values (*p *< 0.001). The participant (random effect) had a variance (SD) of 224.1 (14.97) and contributed significantly to the observed total variance (77%), possibly due to the different CHD subtypes.

Fig. 2C, D, E depict volumetric visualizations from the SVR 4D flow at systole in a patient with Ebstein anomaly. Three-dimensional rendered magnitude image (Fig. 2C) shows the enlarged right ventricle. Maximum intensity projection of the phase-contrast magnetic resonance angiography (Fig. 2D) and particle traces (Fig. 2E) highlight the outflow tract and the blood flow dynamics in these vessels.

## 5. Discussion and conclusions

In conclusion, this study demonstrated the feasibility of SVR 4D flow CMR and compared it to 2D PCMRI measurements in the great thoracic vessels of pediatric participants with CHD. Mean flows from both techniques showed excellent agreement. SVR 4D flowCMR, which benefited from inflow signal enhancement, provided good endogenous contrast for segmentation and may aid future evaluation of CHD. Despite the promising results from the pipeline, there were limitations in this study. First, some measurements suffered from aliasing and automated unwrapping failed. Second, 2D PCMRI was not obtained clinically in all vessels. Third, some vessels suffered from poor coverage in SVR 4D flow CMR due to slice prescription ending prematurely. Fourth, respiratory compensation was not explored in this study. Fifth, long reconstruction times limit the clinical application of SVR 4D flow. However, future computational hardware improvements and machine learning reconstruction strategies may mitigate this limitation. Finally, flow comparisons were limited to 2D PCMRI and SVR 4D flow and did not include conventional Cartesian 4D flow due to clinical scan time constraints. Further research involving a larger cohort of healthy adult participants is warranted to investigate the potential of SVR 4D flow in comparison to conventional 4D flow approaches.

## Funding

Project Grant from the Canadian Institutes of Health Research (PJT 427837) & Discovery Grant from the Natural Sciences and Engineering Research Council (RGPIN-2019–06483) supported data acquisition, result analysis, report writing, and article submission.

## Author contributions

**Valérie Béland:** Writing – review & editing, Writing – original draft, Visualization, Validation, Methodology, Investigation, Formal analysis. **Datta Singh Goolaub:** Writing – review & editing, Writing – original draft, Visualization, Validation, Supervision, Software, Methodology, Investigation, Formal analysis, Data curation, Conceptualization. **Sharon Portnoy:** Writing – review & editing, Methodology, Investigation. **Shi-Joon Yoo:** Writing – review & editing, Investigation, Funding acquisition, Data curation. **Christopher Z. Lam:** Writing – review & editing, Investigation, Funding acquisition, Data curation, Conceptualization. **Christopher K. Macgowan:** Writing – review & editing, Supervision, Resources, Project administration, Methodology, Investigation, Funding acquisition, Conceptualization.

## Ethics approval and consent

This study was approved by our Institutional Review Board, and written informed consent was provided for each participant.

## Declaration of competing interest

The authors declare that they have no known competing financial interests or personal relationships that could have appeared to influence the work reported in this paper.

## Data Availability

The datasets generated in this study were anonymized to respect patient confidentiality and privacy rights and may be available upon request to the corresponding author and approval of the appropriate Research Ethics Board.
